# Antiresonant Hollow-Core Fiber-Based Dual Gas Sensor for Detection of Methane and Carbon Dioxide in the Near- and Mid-Infrared Regions

**DOI:** 10.3390/s20143813

**Published:** 2020-07-08

**Authors:** Piotr Jaworski, Paweł Kozioł, Karol Krzempek, Dakun Wu, Fei Yu, Piotr Bojęś, Grzegorz Dudzik, Meisong Liao, Krzysztof Abramski, Jonathan Knight

**Affiliations:** 1 Laser & Fiber Electronics Group, Faculty of Electronics, Wroclaw University of Science and Technology, Wybrzeze Wyspianskiego 27, 50-370 Wroclaw, Poland; pawel.koziol@pwr.edu.pl (P.K.); karol.krzempek@pwr.edu.pl (K.K.); piotr.bojes@pwr.edu.pl (P.B.); grzegorz.dudzik@pwr.edu.pl (G.D.); krzysztof.abramski@pwr.edu.pl (K.A.); 2 Key Laboratory of Materials for High Power Laser, Shanghai Institute of Optics and Fine Mechanics, Chinese Academy of Sciences, Shanghai 201800, China; wudakun@siom.ac.cn (D.W.); yufei@siom.ac.cn (F.Y.); liaomeisong@siom.ac.cn (M.L.); 3 Center of Materials Science and Optoelectronics Engineering, University of Chinese Academy of Sciences, Beijing 100049, China; 4 Hangzhou Institute for Advanced Study, University of Chinese Academy of Sciences, Hangzhou 310024, China; 5 Centre for Photonics and Photonic Materials, Department of Physics, University of Bath, Claverton Down, Bath BA2 7AY, UK; j.c.knight@bath.ac.uk

**Keywords:** antiresonant hollow core fibers, microstructured fibers, laser spectroscopy, wavelength modulation spectroscopy, fiber gas sensors, fiber optics

## Abstract

In this work, we present for the first time a laser-based dual gas sensor utilizing a silica-based Antiresonant Hollow-Core Fiber (ARHCF) operating in the Near- and Mid-Infrared spectral region. A 1-m-long fiber with an 84-µm diameter air-core was implemented as a low-volume absorption cell in a sensor configuration utilizing the simple and well-known Wavelength Modulation Spectroscopy (WMS) method. The fiber was filled with a mixture of methane (CH_4_) and carbon dioxide (CO_2_), and a simultaneous detection of both gases was demonstrated targeting their transitions at 3.334 µm and 1.574 µm, respectively. Due to excellent guidance properties of the fiber and low background noise, the proposed sensor reached a detection limit down to 24 parts-per-billion by volume for CH_4_ and 144 parts-per-million by volume for CO_2_. The obtained results confirm the suitability of ARHCF for efficient use in gas sensing applications for over a broad spectral range. Thanks to the demonstrated low loss, such fibers with lengths of over one meter can be used for increasing the laser-gas molecules interaction path, substituting bulk optics-based multipass cells, while delivering required flexibility, compactness, reliability and enhancement in the sensor’s sensitivity.

## 1. Introduction

Hollow-core fiber (HCF) technology has experienced rapid and persistent development since the first photonic bandgap HCF was reported by Cregan et al. in the previous century [1]. Since that time, many types of HCFs have been designed and fabricated in order to obtain the lowest possible loss and single-mode guidance, while delivering desired versatility and reliability [2,3,4,5,6]. Due to exceptional light guidance mechanisms present in HCFs, allowing for efficient transmission of laser radiation in the air core, these fibers have found many interesting applications, out of the scope of conventional solid core fibers. The most interesting amongst many are high energy pulsed light delivery [7], pulse compression [8], gas-based lasers [9], telecommunication [10], high harmonic generation [11], and sensing [12] particularly aimed at gas molecules detection [13]. Despite being fabricated from high purity silica glass, such fibers (dependent on the type) enable low-loss light propagation in both Near-(NIR) [13,14] and Mid-Infrared (MIR) [15] spectral regions, where the majority of gases hazardous to human life and environment have their strong molecular fingerprints [16]. With proper design of the sensor, laser gas spectroscopy techniques can take advantage of the guidance performance of the HCFs and the air-core structure, which can be filled with any gas sample (single gas or a gas mixture). Therefore, low-volume fiber-based gas absorption cells providing optical path-lengths (gas molecules—laser light interaction path) precisely fitted to the specific application can be constructed. As the majority of laser-based gas sensors experience significant improvement in their detectivity due to the increased optical path lengths, thus solutions addressing this issue are of great interest to the laser spectroscopy community. Commonly used approaches rely on incorporating bulk-optics-based multipass cells into the laser gas spectrometers [17]. Although the mutlipass cells provide the required interaction lengths, their incorporation significantly affects the long-term stability of a sensor due to their high sensitivity to the influence of temperature changes or vibrations and the overall increase in the detector complexity. Even the application of recently proposed toroidal-type multipass cells does not solve the issues, as such cells require a complicated arrangement of coupling optics to be used, regardless of their compact size [18]. A promising solution to the abovementioned problems comes with the application of HCFs.

First demonstration of the implementation of HCFs into laser-based gas sensors was relying on the use of photonic bandgap HCFs (HC-PBGFs) and enabled detection of various gas molecules in the NIR and MIR [19,20]. Unfortunately, their performance was strongly limited by the multimode nature of the fibers and relatively high loss in the MIR [20,21]. Therefore, effective use of the HC-PBGFs in laser spectroscopy applications entails utilizing complex gas sensing techniques, e.g., Chirped Laser Dispersion Spectroscopy (CLaDS) or photothermal spectroscopy [21,22], hence limiting the required versatility. However, as the HC-PBGFs have physical and optical parameters (dimensions, mode field diameter and numerical aperture) similar to conventional solid core single-mode fibers it is possible to efficiently splice them together [22]. Therefore, optics-free and low-loss light coupling into the HC-PBGF-based absorption cells is obtainable [22]. Another type of HCFs, the so-called hypocycloid core Kagome fiber has been recently used as an absorption cell to target methane transitions in the Mid-Infrared region [23,24]. The proposed Kagome-fiber-based gas sensor utilized the Wavelength Modulation Spectroscopy (WMS) technique and was characterized by significantly better detection limit (at sub-parts-per-million by volume level) in comparison with similar configurations based on the HC-PBGFs [23]. However, as the fiber supported transmission of the higher order modes, CLaDS approach had to be used in order to minimize their impact and reach the maximum detection capacity of the sensor [24]. The next type of hollow-core fibers are the Antiresonant Hollow-Core Fibers (ARHCFs), which have been demonstrated to enable detecting gas molecules at the concentration levels from sub-parts-per-million by volume (ppmv) down to a parts-per-billion by volume (ppbv) levels with the aid of WMS [25,26,27]. Furthermore, ARHCFs and Kagome fibers due to their large core size (in the range from a few ten to over a hundred of µm in dia.) lead to significantly better filling time of the fiber-based gas cell in comparison with HC-PBGFs-based ones [21,23,27]. However, their full potential and versatility, in particular resulting from ability to guide light in several transmission bands, has not been fully explored yet.

In this paper, we demonstrate simultaneous detection of carbon dioxide and methane inside a self-fabricated ARHCF fully benefiting from its low-loss transmission bands covering 1.574 µm and 3.334 µm wavelengths, corresponding to the absorption features of the selected gases. The proposed gas sensor configuration beats the performance of other fiber-based gas detection approaches previously reported utilizing a simple WMS method targeting the aforementioned molecules and strongly emphasizes its versatility.

## 2. Materials and Methods

### 2.1. Antiresonant Hollow-Core Fiber

**Figure 1:**
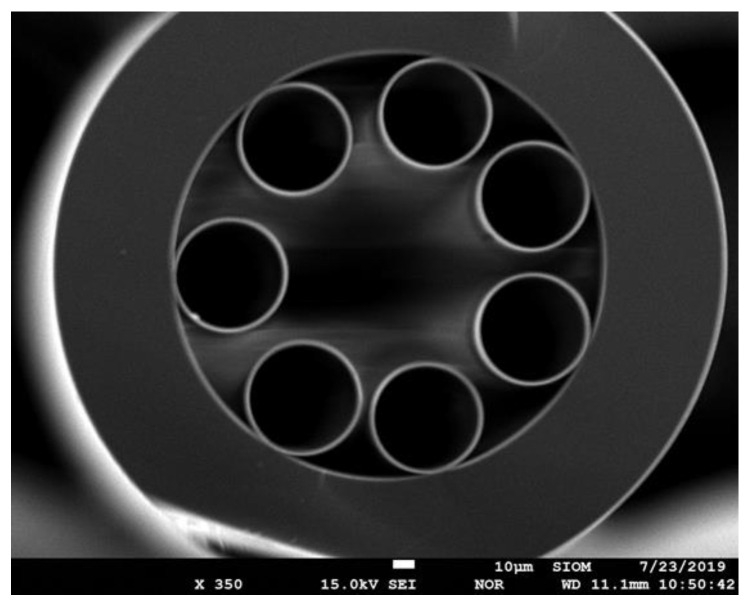
A SEM cross-section image of the fabricated Antiresonant Hollow-Core Fiber (ARHCF).

The ARHCF used in the experiments was made of high purity fused silica glass (Suprasil F300) and fabricated using the common stack-and-draw technique [28]. The capillaries in the cladding were pressurized to prevent them from collapsing during the fiber fabrication. Optimized fiber drawing process enabled us to draw down the final fiber whose cladding structure consists of seven non-touching circular capillaries with outer diameter of d ~55 µm. Surrounded by these cladding capillaries, a central hollow core with a diameter of D ~84 µm was formed. The outer diameter of the ARHCF is 318 µm. A scanning electron micrograph (SEM) of the fiber cross section is depicted in Figure 1.

**Figure 2:**
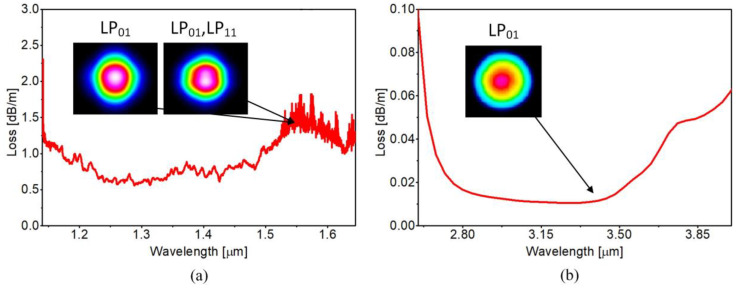
Attenuation spectra of the fabricated ARHCF within: (**a**) Near-Infrared (measured); (**b**) Mid-Infrared (simulated) spectral regions. Near-field fiber-delivered beam profiles captured at 1.574 µm (LP_01_ and mixture of LP_01_ and LP_11_ modes) and 3.334 µm (LP_01_ mode) are presented as insets.

The light guidance mechanism in an ARHCF can be simply explained by the so-called Antiresonant Reflective Optical Waveguiding principle (ARROW). According to ARROW model, the core boundary layer is regarded as Fabry–Perot resonator, supporting the transmission of optical frequencies which match the anti-resonance of the core wall [29]. The resonant wavelengths cannot therefore propagate in the core and leak away to the cladding, experiencing high leakage and material attenuation. The resonant frequency is defined mainly by the core wall thickness (capillary thickness), which in the case of the self-fabricated fiber was ~1 µm. The above parameters enabled broadband guidance in the Near- and Mid-Infrared spectral regions, respectively as shown in Figure 2.

In the NIR the fiber is characterized by the loss of ~0.5–1.5 dB/m, while in its fundamental band in the MIR the performed simulations showed that the loss characteristic of the fabricated fiber is significantly lower. The exact loss in the MIR was calculated using the cutback technique at 3.334 µm wavelength with an aid of a custom built DFG (difference frequency generation) system used as a coherent light source (described in details in Section 2.2). The fiber was cut from 20 m to 1.5 m maintaining the same coupling conditions. The loss was calculated as a difference between the optical powers measured at the output of the fiber before and after the cutback was performed. The calculated loss was 0.03 dB/m. The difference between the loss measured in the NIR and MIR is mainly connected with the greater bend sensitivity of the fiber at shorter wavelengths due to the too large core size for the shorter wavelength range. It was shown in [30] that the core size, hence the ratio d/D has a direct impact on the bend loss. As the structure was optimized to obtain d/D ~0.65, the ARHCF exhibits good single-mode guidance over the transmission bands because of increased loss ratio of fundamental and higher-order modes [30]. Of course, our measurement shows that the ARHCF supports both LP_01_ and LP_11_ modes in the Near-Infrared spectral region, which is possibly due to the significantly larger core size diminishing the loss difference between fundamental and higher-order modes at shorter wavelengths. Near-field fiber-delivered beam profiles showing supported modes within both transmission bands were captured by directly imaging the fiber end-facet onto a scanning slit optical beam profiler (model BP209-IR, Thorlabs GmbH, Bergkirchen, Germany) and a microbolometer (model WinCamD16, DataRay, Redding, USA), and are shown as insets in Figure 2a,b. Excitation of the higher order modes within the fundamental low loss band of the fiber did not occur.

### 2.2. Experimental Setup

**Figure 3:**
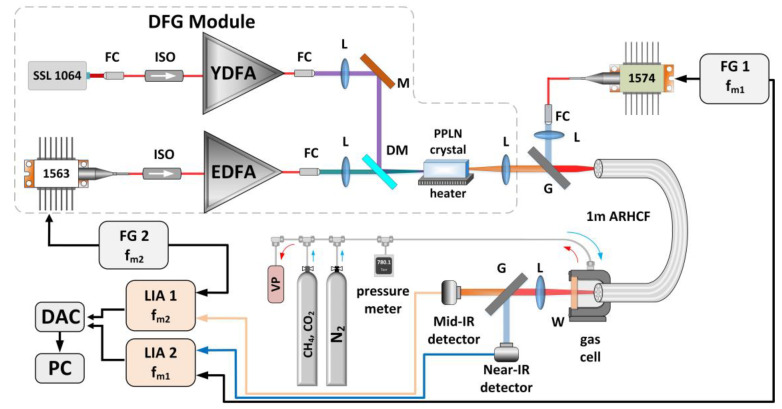
Schematic of the experimental setup. FG 1 and FG 2—function generators, DFG—difference frequency generation source, f_m1_ and f_m2_—modulation frequencies, L—lenses, G—germanium window, ARHCF—Antiresonant Hollow-Core Fiber, W—calcium fluoride wedge, VP—vacuum pump, LIA 1 and LIA 2—lock-in amplifiers, DAC—data acquisition card, PC—laptop computer, SSL 1064—solid state laser, 1563 and 1574—DFB lasers, FC—fiber collimator, ISO—fiber isolator, EDFA—erbium doped fiber amplifier, YDFA—ytterbium doped fiber amplifier, DM—dichroic mirror.

A schematic representation of the experimental setup is presented in Figure 3.

**Figure 4:**
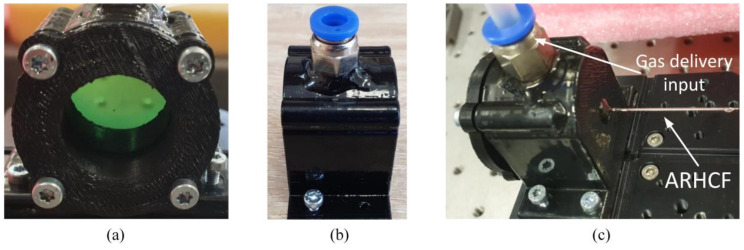
Photographs of a 3D-printed airtight housing used for filling the ARHCF core with the target gas mixture. (**a**) Front-view. CaF_2_ wedge is used to enable out-coupling of the fiber-delivered beam from the housing, providing undisturbed gas flow. (**b**) Side-view with a pneumatic inlet mounted on top for efficient gas delivery. (**c**) The housing mounted on a XYZ translation stage with the ARHCF glued into it (parallel to the central axis of the housing) with a UV-curing glue via a glass capillary with an internal diameter of 500 µm, preventing the fiber from unintentional bending inside the housing. The gas mixture was delivered through a 6 mm diameter tube. The housing was experimentally tested to withstand a pressure of 2.5 bar (~1875 Torr).

To efficiently excite molecules of the target gases—CO_2_ and CH_4_, two coherent, narrow-linewidth light sources were used. CO_2_ was probed with a distributed feedback diode laser (DFB, model NLK1L5EAAA, NTT Electronic, Yokohama, Japan) operating at 1.574 µm (6353.1 cm^−1^) and delivering ~20 mW of optical power, for which the operating current and temperature set points (17.2 °C) were driven by a commercially available controller ( model ITC4020, Thorlabs GmbH, Bergkirchen, Germany). CH_4_ molecules were excited at 3.334 µm (2999.01 cm^−1^) using a custom-made MIR laser based on a difference frequency generation phenomenon (DFG) [31], as depicted in Figure 3. The developed DFG source utilized a home-built diode-pumped solid-state Nd:YVO_4_ laser delivering 40 mW CW single-mode radiation at 1064.3 nm and a DFB fiber-pigtailed laser diode providing tunable output in the vicinity of 1563 nm (model QDFBLD-1560-20, QPhotonics, Ann Arbor, USA), which was driven and temperature stabilized (at 28.2 °C) by a commercial controller (model CLD1015, Thorlabs GmbH, Bergkirchen, Germany). Both seeds emitted linearly polarized beams. The seed beams were amplified in custom built, two-stage all-PM-fiber amplifiers to maximize the DFG-delivered optical power. After amplification, the 1064.3 nm and 1563 nm beams reached the optical powers at the level of ~1.2 W each. The amplified NIR beams were co-axially combined and subsequently focused using bulk optics elements into a 40-mm-long PPLN crystal (periodically poled lithium niobate, model MOPO1-1.0-40, Covesion Ltd., Romsey, UK) in order to generate MIR light at the wavelength corresponding to the center of the target methane transition. The PPLN crystal was kept at a constant temperature of 93.2 °C using a temperature controller (model OC1, Covesion Ltd., Romsey, UK), which ensured optimum quasi phase matching conditions. As a result, the DFG process provided a CW (continuous wave) output with ~0.5 mW optical power. Gas molecules excitation beams were co-axially combined using a germanium window and simultaneously coupled into the ARHCF core using plano-convex calcium fluoride (CaF_2_, focal length of f = 75 mm) and N-BK7 (f = 100 mm) lenses with proper anti-reflective coatings for 3.334 µm and 1.574 µm, respectively. Both lenses were carefully selected in order to match the numerical aperture (NA) of the fiber [32]. One end of the fiber was glued into a custom-made 3D-printed airtight housing sealed with a CaF_2_ wedge, which was used to fill the fiber core with the mixture of 100 ppmv CH_4_ and 10% of CO_2_ at the constant pressure of 800 Torr (Figure 4). The beams exiting the ARHCF were collimated using a single CaF_2_ plano-convex lens (f = 75 mm). MIR and NIR fiber-delivered beams were separated on a germanium window and subsequently focused with an aid of CaF_2_ and N-BK7 plano-convex lenses (both with f = 25.4 mm) onto the facets of mercury–cadmium–telluride (MCT, model PVI-4TE-8VPAC-1000F, Vigo System S.A, Ozarow Mazowiecki, Poland) and indium–gallium–arsenide (InGaAs, model PDA10DT, Thorlabs GmbH, Bergkirchen, Germany) photodetectors, respectively. Electrical signals produced by both photodetectors were fed to two lock-in amplifiers (LIA, model SR830, Stanford Research System, Sunnyvale, USA), each set to analyze different frequency components. The signals from LIAs were digitized and visualized with the aid of a data acquisition card (model USB-4432, National Instruments, Austin, USA) combined with a standard laptop PC.

**Figure 5:**
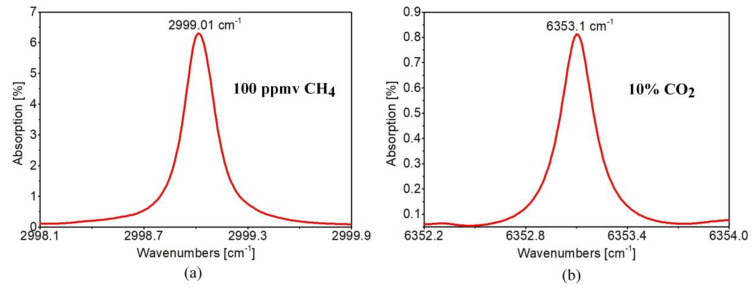
Absorption spectra of target transitions of: (**a**) 100 ppmv CH_4_; (**b**) 10% CO_2_ both simulated for 1 m path length and 800 Torr pressure using the HITRAN database [34].

The laser spectroscopy method implemented in the proposed sensor to detect both gases was WMS [33]. In WMS, the wavelength of the laser that is used to probe gas molecules is modulated with a sinusoidal signal with a defined amplitude and frequency (f_m_), in our case through injection current delivered to the lasers. Interaction of the modulated laser radiation with a gas transition results in the rise of additional signals at harmonics of the f_m_, i.e., 2xf_m_, 3xf_m_, etc., that can be retrieved from the detected electrical signal using a lock-in-based data processing approach. The amplitude of even WMS signal harmonics is proportional to the molecular concentration, hence enabling this technique to be used as a precise tool for detecting gas species. In the proposed sensor configuration, the CH_4_ and CO_2_ probing lasers wavelengths were modulated with a sine-wave current signals at f_m1_ = 1 kHz (applied to 1563 nm DFB seed laser from the DFG) and f_m2_ = 2.1 kHz, respectively using arbitrary function generators (FG, model AFG3102C, Tektronix, Inc., Beaverton, OR, USA). Additional low-frequency (100 mHz) saw-tooth ramp signals were applied to the current of each laser using FGs in order to retrieve full second harmonic WMS signal spectra of the selected CH_4_ and CO_2_ transitions (as shown in Figure 5).

## 3. Results

### 3.1. Sensor Characterization

**Figure 6:**
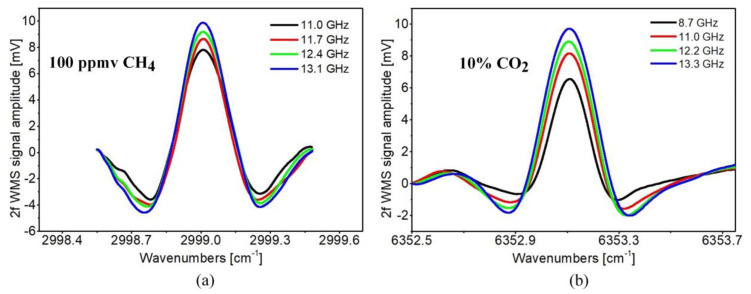
2f wavelength modulation spectroscopy (WMS) signal spectra retrieved for different wavelength modulation amplitudes of the Near- (NIR) and Mid-Infrared (MIR) lasers from: (**a**) 100 ppmv CH_4_; (**b**) 10% CO_2_ inside a 1-m-long ARHCF. Please note that the signals produced by both gases were recorded for different sensitivity settings of the LIAs used for each gas (with an order of magnitude of difference), hence their amplitude level cannot be directly compared.

In order to eliminate ambient air from the air core of the ARHCF (which may introduce parasitic background signal due to the atmospheric concentration of the selected gas molecules), the fiber was firstly rapidly flushed with pure nitrogen (N_2_) at the pressure of 1200 Torr and subsequently filled with a calibrated mixture of the target gases at a slight and well-controlled pressure of 800 Torr (set at the fiber input). Under such conditions the gas flow was maintained undisturbed along the entire fiber length within the measurements duration. The operational parameters of the proposed sensor, which enabled efficient second harmonic WMS signal retrieval from both gases, were determined experimentally. The LIAs were set to detect signals at the frequencies of 2 kHz and 4.2 kHz (corresponding to the second harmonics of the sinusoidal modulation frequencies applied to the probe lasers) for CH_4_ and CO_2_ measurements, respectively. Recorded 2f WMS signals for 100 ppm of CH_4_ and 10% of CO_2_ flowing through the ARHCF core are shown in Figure 6. The optimum wavelength modulation amplitudes reached 13.1 GHz for methane and 13.3 GHz for carbon dioxide. Due to unavailability of a seed laser emitting 1563 nm radiation that enables wider wavelength tunability it was impossible to record 2f WMS signal from CH_4_ over a broader range. The main source of the background fringes originated from the PPLN crystal itself, due to interference on its flat input and output surfaces. The impact of the coupling between LP_01_ and LP_11_ modes in the NIR, and hence unwanted intermodal interference can be observed on the 2f WMS signals from CO_2_ (especially visible on the shorter wavenumber range in Figure 6b). Such phenomenon is typical for the fiber-based gas sensors utilizing multimode guiding fibers [21].

**Figure 7:**
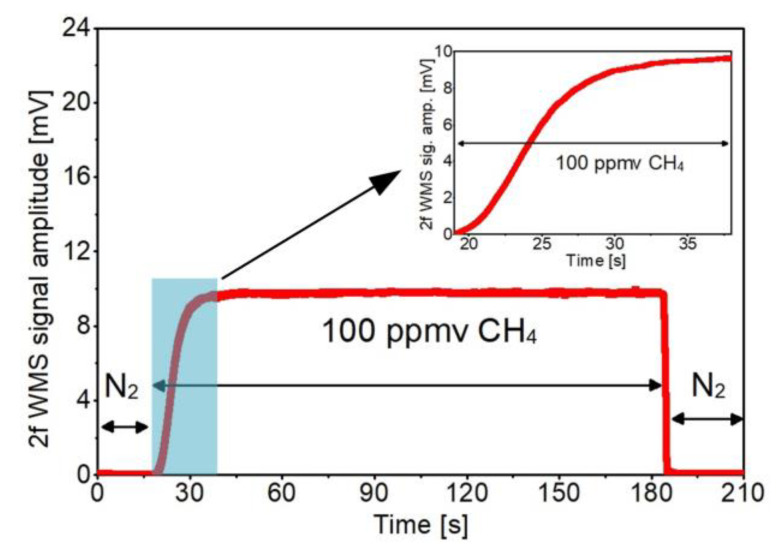
Gas filling time determined by measuring 2f WMS signal amplitude while the ARHCF fiber was subsequently flushed with pure N_2_ and a mixture of 100 ppmv CH_4_ and 10% CO_2_ using pressures of 1200 Torr and 800 Torr (at the fiber input), respectively. The measurements and calculations were performed for CH_4_ using a 3.334 µm DFG laser set the absorption peak of the gas.

The filling time of the proposed ARHCF-based gas sensor was established by measuring the time required for filling the fiber core with CH_4_. The operating parameters of the DFG source were set to deliver radiation at the wavelength corresponding to the center of the selected CH_4_ transition, without any additional active stabilization. The fiber was firstly purged with pure N_2_ using 1200 Torr pressure (at the fiber input), so only the background signal could be registered. Next, we started to fill the fiber with a mixture of 100 ppmv CH_4_ and 10% CO_2_ at a pressure of 800 Torr (measured at the fiber input), and the amplitude of 2f WMS signal was registered. As presented in Figure 7, the amplitude of the 2f WMS signal reached 90% of its maximum value after ~19 s, which corresponds to the gas flow rate at the level of 17.5 µl/min. At the end of the measurement, the fiber was again rapidly flushed with N_2_ (at 1200 Torr pressure) and target gases molecules were evacuated from the fiber core.

### 3.2. Sensor Performance

**Figure 8:**
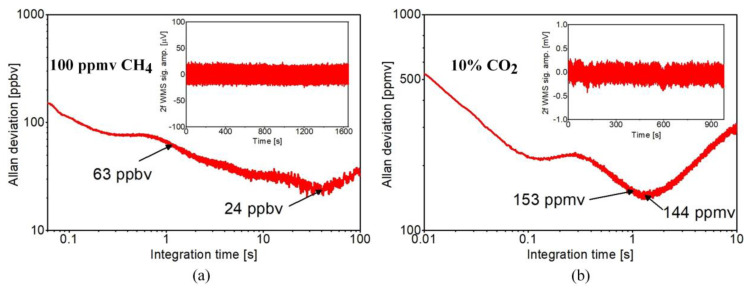
Allan–Werle plots estimating the performance of the sensor in detecting CH_4_-(**a**) and CO_2_-(**b**). The plots were calculated based on 2f signal registered for pure N_2_ flushed into the fiber and both excitation lasers tuned to the center of the gas transitions (insets in both graphs). The wavelength of the lasers was not actively locked.

The minimum detection limits (MDL) of the proposed ARHCF-based dual gas sensor were established experimentally by analyzing the senor’s long-term stability in the MIR and NIR spectral ranges. The method of estimating the MDL was realized according to the reference [35]. Firstly, the fiber was filled entirely with a mixture of 100 ppmv CH_4_ and 10% CO_2_ at 800 Torr pressure (at the fiber input). The parameters of both laser sources were set to operate at the optimum conditions, so the amplitude of the registered 2f WMS signal for both gases reached their maximum levels (according to results shown Figure 6). The central wavelengths emitted by the excitation sources were set to the centers of the selected gases transitions. The wavelengths of the DFG and DFB sources were not additionally stabilized within the measurements duration. In the second step, the fiber core was flushed with pure N_2_ (at 800 Torr pressure set at the fiber input) and the 2f WMS signal amplitudes for both laser sources were recorded with an acquisition rate of 1 kHz. The time constant for the LIA was set to 1 ms with a filter slope of 18 dB/oct. The recorded results are plotted as the insets in Figure 8. The MDLs of the proposed sensor configuration were calculated based on the Allan–Werle plots obtained from these data sets and are shown in Figure 8a,b, for the sensor targeting CH_4_ and CO_2_, respectively [36]. For 1 s averaging time the MDLs reached 63 ppbv for CH_4_ and 153 ppmv for CO_2_. For 40 s averaging time the MDL for methane reached ~24 ppbv, while 144 ppmv MDL was obtained at approximately 1.5 s integration time for carbon dioxide. No further improvement in the sensor’s sensitivity was observed for longer averaging times for the CO2 detection due to non-optimal transmission characteristic of the ARHCF at this wavelength, resulting in intermodal interference. The obtained MDLs correspond to the minimum detectable fractional absorption (MDFA) of ~1.6 × 10^−5^ and 1.17 × 10^−5^ for CH_4_ and CO_2_, respectively (calculated based on the data from HITRAN database for 24 ppbv CH_4_ at 2999.01 cm^−1^ and 144 ppmv CO_2_ at 6353.1 cm^−1^ within 1 m path length).

## 4. Discussion

In this paper, we reported on the first experimental verification of the possibility of dual-band gas detection in a single ARHCF. We have demonstrated simultaneous detection of two dissimilar gases by targeting their transitions in the Near- and Mid-Infrared spectral regions. To date, the experimental results published on hollow-core fiber-assisted sensing of carbon dioxide and methane are mainly focused on utilizing well-known and commercially available hollow-core photonic bandgap fibers. In the gas sensor published in [19] a HC-PBGF operating in the vicinity of 2 µm wavelength range was used to detect CO_2_, while accessing very strong transitions of this particular gas in the NIR band. That configuration reached a minimum detectable concentration of CO_2_ equal to 2%, which is far above the ambient level of this gas (~400 ppmv). The result is almost 140 times worse than that achieved in our sensor, which operated in the wavelength range of 1.574 um, where CO_2_ has approximately 80 times lower absorption in comparison with the 2-µm band. In [19], the main limiting factor for the performance of the sensor was the type of the fiber used as the low-volume gas cell (in particular its guidance mechanism) and its relatively short length, which resulted in a strong impact of intermodal interference. A multimode characteristic of the HC-PBGFs is the main source of the background noise, which significantly limits the performance of such gas sensors. In order to minimize the impact of this parasitic effect, more complex spectroscopic methods need to be used. One such techniques is CLaDS, which allows for reduction of intermodal interference, which was demonstrated in [21], as a HC-PBGF-based gas sensor. That CLaDS-based sensor was used to detect carbon dioxide at 1572 nm and yielded a MDL of 422 ppmv, which is approximately three times worse than what was obtained in the case of the presented ARHCF-based sensor aided with less-complex WMS method. Since our fiber is characterized by a few modes of behavior and operates in its second, non-optimized transmission band, a significant improvement in the detection capacity is expected in a modified fiber design, enabling single-mode propagation within its both low-loss windows. Furthermore, due to a very small core size (~10–20 µm in dia.) HC-PBGFs-based gas sensors are characterized by a very slow gas filling time in the range of 20 min (for 1-m-long fiber), which is more than 60 times slower in comparison with the 19 s filling time reported within this work. HC-PBGFs were also implemented in laser-based gas sensors targeting CH_4_ transitions at ~1.65 µm and 3.1 µm [20,37,38]. The best results using a HC-PBGF as an absorption cell were reported in [37]. Here, the light coupling conditions into a hollow-core fiber, proper fiber-length and CH_4_ transition selections enabled both minimizing the impact of intermodal interference, hence reducing the amplitude of the parasitic signal modulation and observing a decrease in noise level. As a result, the obtained MDL was increased to 360 ppbv for 75 s averaging time. This is more than an order of magnitude worse than that presented in this work, based on a single-mode ARHCF designed for the 3.334 µm wavelength range (absorption level of 100 ppmv CH_4_ at 3.334 µm is comparable with the absorption observed by the Authors in [37], hence both results can be compared). In [20], the Authors demonstrated CH_4_ detection around 3.1 µm wavelength using a HC-PBGF. Due to relatively strong light-glass overlap present inside such fibers (limiting the possibility for efficient light guidance in the Mid-Infrared, where strong glass absorption is experienced by the laser radiation) and multimode fiber characteristics, the proposed sensor’s sensitivity at optimum operation conditions was at the level of 50 ppmv. The obtained MDL was more than three orders of magnitude less than that obtained in this work.

Recently, a Kagome-type hollow-core fiber designed to guide light within 3.4 µm wavelength band was implemented in a WMS-based sensor configuration aimed on detection of methane at 2999 cm^−1^ [23]. A 1.3-m-long fiber with a ~100 µm air core was used as an absorption cell and yielded a MDL and MDFA of 0.5 ppmv and 4.5 × 10^−4^, respectively. The detection capacity of the Kagome fiber-based gas sensor in comparison with our sensor was more than an order of magnitude worse, mainly due to the multimode guidance of the Kagome fiber, which resulted in the presence of unwanted signal modulation increasing the system’s noise. The same Kagome fiber was also used in combination with CLaDS technique in order to minimize the influence of intermodal interference and obtain improved sensitivity of the CH_4_ sensor [24]. The reported sensor obtained a MDL of 65 ppbv and MDFA of 5.2 × 10^−5^, which is still inferior to the ARHCF-based sensor described in this work, utilizing a significantly simpler sensing approach and a less complex fiber structure. Gas sensing in an ARHCF was reported in [39]. The constructed sensor was based on a 1.35-m-long revolver-type antiresonant fiber (similar to our fiber) with a core diameter of 70 µm, which combined with WMS was used to target strong CO_2_ transition at 2004 nm. The presented 2f WMS signals clearly indicated its multimodal nature, which resulted in the presence of a parasitic modulation signal. As a result, the MDFA reached the level of 1 × 10^−4^, which was approximately an order of magnitude worse than the level presented in the dual-band ARHCF-based gas sensor presented in this work.

The measured gas exchange time of our sensor is approximately five times longer than what has been reported previously with Kagome-type or other ARHCF fibers with larger core sizes and similar lengths. However, previous experiments required using higher over-pressures to exchange the gas in the fiber, thus producing pressure-broadened absorption profiles [23,27,39]. Nevertheless, the 19-s exchange time achieved in our configuration is acceptable for various gas sensing applications.

The reported dual-band dual-gas ARHCF-based gas sensor reached MDFA of ~1.6 × 10^−5^ and 1.17 × 10^−5^ for CH_4_ and CO_2_, respectively, which is better compared to previously published sensors utilizing the non-complex WMS detection technique.

## 5. Conclusions

The performed experiments proved that the multi-band low-loss transmission of the ARHCFs can be conveniently used as an advantage, enabling simultaneous and efficient detection of dissimilar gases with their absorption features both in the Near- and Mid-Infrared spectral regions. The main advantage of using ARCHFs as gas absorption cells is connected with their unique guidance mechanism resulting in multiple and broad transmission bands (their position can be adjusted via fiber structure modification), which cover the spectral ranges where various gases have their strong molecular fingerprints. The large core size is another advantage of this particular fiber as it enables relatively easy and fast gas filling time, which can be significantly reduced by introducing a proper modification of the gas filling approach (e.g., gas pumping into the core via micro-channels along the fiber length). Finally, such fibers with several meters to several tens of meters in length can provide the performance in terms of application into gas sensing at the level overcoming the performance delivered by multipass cells. The presented work indicates that further development and optimization of such fibers is crucial and will definitely benefit the future development of versatile, compact, selective and sensitive laser-based gas sensors, eliminating the requirement of using fragile bulk optics-based absorption cells.
